# Leveraging local ancestry to detect gene-gene interactions in genome-wide data

**DOI:** 10.1186/s12863-015-0283-z

**Published:** 2015-10-24

**Authors:** Hugues Aschard, Alexander Gusev, Robert Brown, Bogdan Pasaniuc

**Affiliations:** Department of Epidemiology, Harvard School of Public Health, Boston, MA USA; Bioinformatics Interdepartmental Program, University of California Los Angeles, Los Angeles, CA USA; Department of Pathology and Laboratory Medicine, University of California Los Angeles, Los Angeles, CA USA; Department of Human Genetics, University of California Los Angeles, Los Angeles, CA USA

**Keywords:** Gene-gene interaction, GWAS, Local ancestry, Statistical genetics

## Abstract

**Background:**

Although genome-wide association studies have successfully identified thousands of variants associated to complex traits, these variants only explain a small amount of the entire heritability of the trait. Gene-gene interactions have been proposed as a source to explain a significant percentage of the missing heritability. However, detecting gene-gene interactions has proven to be very difficult due to computational and statistical challenges. The vast number of possible interactions that can be tested induces very stringent multiple hypotheses corrections that limit the power of detection. These issues have been mostly highlighted for the identification of pairwise effects and are even more challenging when addressing higher order interaction effects. In this work we explore the use of local ancestry in recently admixed individuals to find signals of gene-gene interaction on human traits and diseases.

**Results:**

We introduce statistical methods that leverage the correlation between local ancestry and the hidden unknown causal variants to find distant gene-gene interactions. We show that the power of this test increases with the number of causal variants per locus and the degree of differentiation of these variants between the ancestral populations. Overall, our simulations confirm that local ancestry can be used to detect gene-gene interactions, solving the computational bottleneck. When compared to a single nucleotide polymorphism (SNP)-based interaction screening of the same sample size, the power of our test was lower on all settings we considered. However, accounting for the dramatic increase in sample size that can be achieve when genotyping only a set of ancestry informative markers instead of the whole genome, we observe substantial gain in power in several scenarios.

**Conclusion:**

Local ancestry-based interaction tests offer a new path to the detection of gene-gene interaction effects. It would be particularly useful in scenarios where multiple differentiated variants at the interacting loci act in a synergistic manner.

**Electronic supplementary material:**

The online version of this article (doi:10.1186/s12863-015-0283-z) contains supplementary material, which is available to authorized users.

## Background

Advances in high-throughput genotyping technologies have enabled large-scale studies of genetic variation, from genome-wide association studies (GWAS) to inference of population history. The most notable use of high-throughput genotyping has been in GWAS where researchers have reproducibly identified thousands of genetic variants associated with many complex traits and common diseases. Despite the great success in identifying variants that contribute risk to disease, the majority of the genetic component of human traits and diseases remains unexplained. A potential source for this missing heritability is gene-gene interactions that alter disease risk in a coordinated fashion, for example when several genes are acting synergistically on a trait. Although of potential great interest, robust identification of gene-gene interactions has largely remained elusive, and despite numerous studies only a few interaction effects have been detected in human data [1–3]. Most genetic association studies of gene-gene interaction have focused on the joint effect on pairs of single nucleotide polymorphisms (SNPs) and used brute force approaches to evaluate a large number of pairs on homogenous populations (e.g. individuals of European ancestry only), while alternative strategies using heterogeneous populations have been seldom considered [4, 5].

The development of accurate methods for discerning population structure have allowed for studies across different ethnicities including admixed populations (i.e. populations with recent ancestry from more than one continent such as African Americans). In addition to the standard linkage disequilibrium (LD) between nearby markers (used by GWAS to tag hidden causal variants) admixed populations exhibit another form of correlation among variants at a coarser scale due to chromosomal segments of distinct ancestry that is commonly referred to as admixture-LD [6]. This enables admixture mapping to be an effective approach for identifying disease loci that differ in frequency across populations [7–11]. A key component of such studies is the inference of ancestry at each locus in the genome. Several computational and statistical tools, including HAPMIX [12], LAMP-LD [13], EILA [14], and LANC-CSVs [15] can now be used to reliably call local ancestry. Although local ancestry has been traditionally used in admixture mapping, recent works use analyses of local ancestry to yield novel insights into the dynamics of recombination rate across the genome, to make demographic inferences from genetic data of admixed populations, as well as to understand the genetic basis of complex traits [16–18].

In this work we explore the use of local ancestry in recently admixed populations to find signals of gene-gene interaction that affect disease risk. We introduce an approach that leverages the correlation between local ancestry and the hidden unknown causal variants to find distant –e.g. on different chromosomes– gene-gene interactions. Our proposed approach uses multiple linear regression to model the interaction effect between pairs of local ancestry segments. Hence, as opposed to the standard approaches that test all pairs of SNPs assayed in GWAS (e.g. on the order of 10^12^ pairs for a standard GWAS of 2.5 million SNPs), we propose to test interaction only between pairs of local ancestries (on the order of 5×10^5^ pairs for recent admixtures). By performing a much smaller number of statistical tests, our approach solves the computational bottleneck and reduces the multiple testing correction burden. We derive the analytical formulation for our test assuming a single causal variant for each interacting locus and investigate its performance across a wide range of parameter values. Motivated by recent works that show ever-increasing evidence for multiple causal variants per locus [19–21], we extend our approach to allow for multiple causal variants at each interacting locus. We find that local ancestry can be used to find gene-gene interactions, with power increasing with the number of causal variants per locus and the degree of differentiation in the frequency of the causal variants between the ancestral populations. Assuming equal sample size, the test based on pairwise genotyped SNPs appears to be more powerful than the ancestry-based interaction test in most scenarios. However, when accounting for the increase in sample size that can be achieved for a fixed budget when measuring local ancestry only (e.g. based on ancestry informative markers, AIMs), we observed a substantial increase in power under various scenarios.

## Results and discussion

### Overview of the approach

A standard approach for finding pairwise SNP interactions is to test for non-zero effect size of the product term of the two SNPs considered. The underlying assumption is that the SNPs tested in the model are either the interacting causal variants or correlated to the actual causal variants through LD. Indeed, only a finite number of SNPs are assayed in GWAS (today’s genotyping arrays assay a few million SNPs), with true biologically causal variants likely remaining untyped. While a number of additional SNPs can be imputed on a genome-wide scale, the presence of the causal variants in the data can only be assumed for whole-genome sequence data. It is likely the causal variants will only be tagged by the SNPs analyzed. In admixed populations, correlation between SNPs also exists at a coarse level due to the segments of recent ancestry (admixture-LD). Similar to the pairwise SNP interaction screening, we can tag the hidden causal variants using admixture-LD and test for the presence of interaction at hidden causal variants by testing for interaction at the level of local ancestry.

### Testing for interaction under a single causal variant per locus assumption

**Fig. 1:**
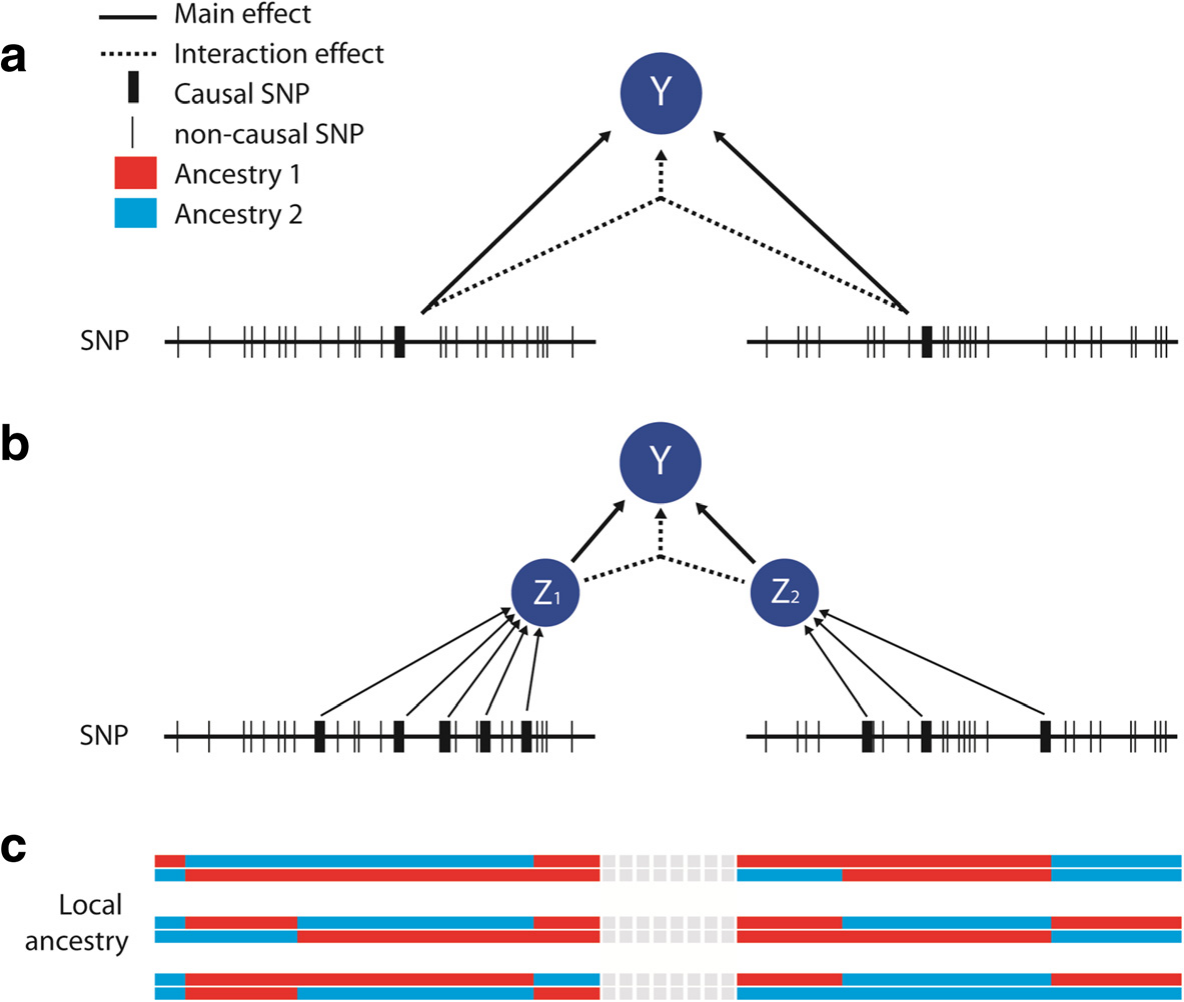
Simulation schemes. Main and interaction effects are simulated assuming either a single genetic variant per locus (**a**) or multiple genetic variants per locus (**b**). In the latter case, the main and interaction effects on the outcome Y are moderated through two latent variables Z_1_ and Z_2_ that directly depend on the causal variants. Example of local ancestry derived for the two haplotypes of three individuals (**c**)

**Fig. 2:**
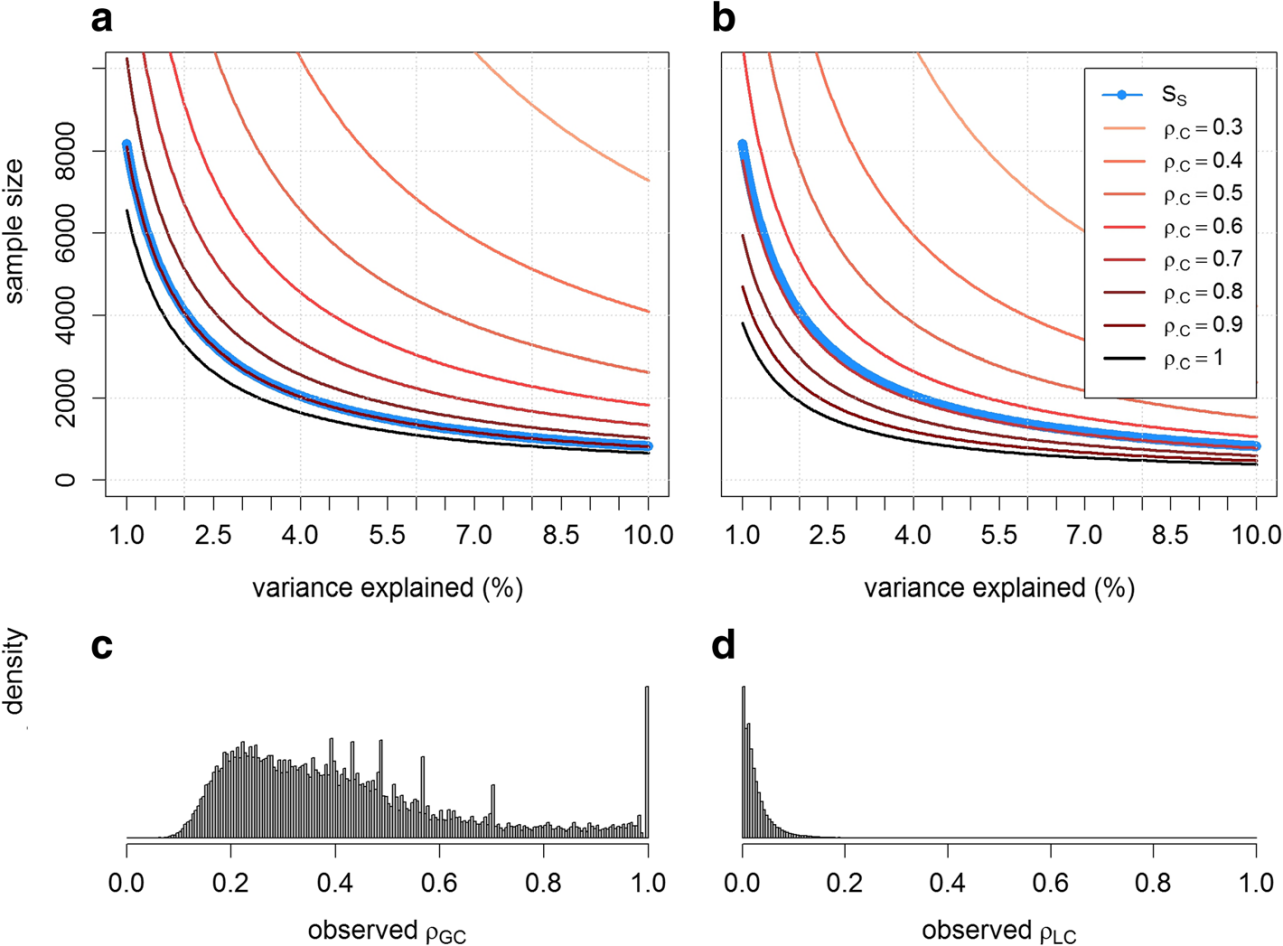
Power comparison for a single causal SNP per locus. Upper panels show the sample size required for 80 % power for the interaction test based on 1 M genotyped GWAS SNPs (*S*_*G*_) (**a**), and the interaction test based on local ancestry segment (*S*_*L*_) assuming a total of 1 K local ancestry segments (**b**) against the interaction test based on full sequencing data (*S*_*S*_) assuming a total of 20 M genetic variants (blue curve). Sample size is plotted for increasing *ρ*_*GC*_ and *ρ*_*GL*_ (defined by the red gradient), the correlation between the true interaction term and the best tag from 1 M genotyped SNPs, and the best tag from local ancestries, respectively. The variance explained by the interaction effect is unrealistically large for illustration purposes and varied between 1 and 10 %. Lower panels show the observed distribution of *ρ*_*GC*_ (**c**) and *ρ*_*GL*_ (**d**) for a randomly selected region from the 1 M Illumina chip and local ancestry, respectively

We first considered a scenario where two common SNPs are located on two physically distant segments in the genome, thus independent from each other, and have an interactive effect on a quantitative phenotype, while all other SNPs at the locus harboring these two causals have no effect (Fig. 1a). We derived the performances of three interaction tests, based on full sequence data (S_S_), genotypic data from a 1 M (1 million) SNPs chip (S_G_) and local ancestry (S_L_). Figure 2 presents the sample size required for each of the three interaction tests to achieve a significance level of 5 % with 80 % power after correction for multiple testing. The sample sizes are plotted for a range of correlation levels between the causal variant and the tagging SNP or tagging ancestry segment. More specifically, we refer to *ρ*_*GC*_ for the correlation between the true interaction term and the interaction term derived from the best tag from the genotyping chip, and to *ρ*_*LC*_ for the correlation between the true interaction term and the local ancestry interaction term between the two segments harboring the causals. For simplicity we assumed here that local ancestry is inferred with high accuracy (*r*^2^ between true ancestry and inferred ancestry ≥0.99), and therefore does not differ from true local ancestry. This will likely be the case for African Americans [15], but might be too optimistic for other populations such as Latino Americans (see below). Figure 2 shows that for GWAS-based test (*S*_*G*_) to outperform the test based on the true causal variants (*S*_*S*_), it requires *ρ*_*GC*_ to be above 0.9. For the local ancestry test, *ρ*_*LC*_ has to be above 0.7. Moreover, for *ρ*_*LC*_ above 0.8, the ancestry based interaction test would also outperform the GWAS-based interaction test even if the causal variants were genotyped. As expected, the maximum potential gain is achieved when the interaction term is perfectly tagged by either GWAS SNPs or local ancestry. We considered unrealistically large interaction effects in Fig. 2 for illustration purposes. When analyzing 20,000 samples, the smallest interaction effect (as measured by the proportion of variance explained) that can be detected with 80 % power is 0.8, 0.5 and 0.3 % for *S*_*S*_, *S*_*G*_ and *S*_*L*_, respectively.

We then estimated the empirical distribution of *ρ*_*GC*_ and *ρ*_*LC*_ using African-American individuals simulated using the 1,000 Genomes data (see Methods) [22]. From this simulation we randomly choose 20,000 independent SNPs, and built 10,000,000 hypothetical pairs of interacting SNPs. Bottom panels of Fig. 2 shows the distribution of these two correlation terms when using tagging SNPs from the 1 M Illumina chip and the simulated local ancestry. Despite the large potential increase in power shown in the upper panels of Fig. 2, improvement may actually exists only in very few real situations. For example we observed that the probability of *ρ*_*GC*_ to be above the 0.9 threshold is 0.05*.* For *ρ*_*LC*_ the “increased power” threshold of 0.7 is achieved only once in 10^7^ times. Hence, even if interaction effects are extremely common in the architecture of complex trait, there is a low probability for the local ancestry–based test to do better than other approaches in the presence of a single causal variant per locus when assuming equal sample size for both tests.

### Multiple causal SNPs per segment

Accounting for increasing evidence of multiple causal variants per locus [19–21], we then considered scenarios where gene-gene interaction effects involved multiple genetic variants per locus. For example when multiple SNPs contribute to gene transcript abundance and the interaction is taking place between the gene products. Such interaction would be challenging to identify using SNP data due to the vast search area among all possible combinations of SNPs. On the other hand, local ancestry offers a more appropriate and natural way to test for such models as it captures a form of an individual’s genetic background at each locus (i.e. genetic variants share the same local ancestry at a given locus in the genome).

**Fig. 3:**
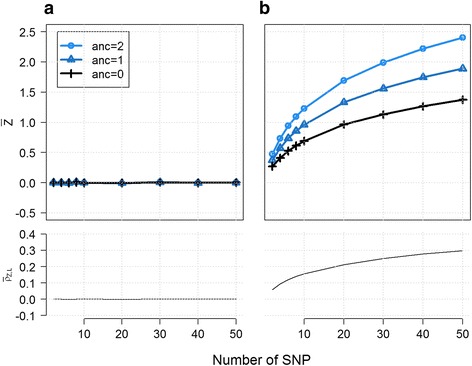
Tagging interaction effects in a multiple causal model. A latent variable *Z* is generated as a function of an increasing number of SNPs at a single locus, explaining altogether 50 % of its variance. The average value of *Z* across 20,000 replicates of 10,000 admixed samples is plotted for each three local ancestry classes. The effect of the SNPs is drawn from a normal (**a**) and left-truncated normal (**b**) distribution with a mean of 0 (upper panel). When the SNP effects are null on average, the average values of *Z* do not differ by local ancestry and *ρ*_*ZL*_, the correlation between *Z* and local ancestry, is also null on average. Conversely, when the average effect of the SNPs is not null, *ρ*_*ZL*_ increases with an increasing number of causal variants (lower panel)

To evaluate this assumption we defined a simulation model where multiple SNPs at two independent loci contribute to two latent variables *Z*_1_ and *Z*_2_ that have an interaction effect on the outcome (Fig. 1b). The power of the pairwise SNPs test depends on the best tagging SNPs for *Z*_1_ at locus 1 and for *Z*_2_ at locus 2. This would be either the strongest causal variants for *Z*_1_ or *Z*_2_, or the best tag of these causals. The power the local ancestry-based test to detect this interaction depends on all parameters influencing ${\rho}_{L_i{Z}_i}$, the correlation between *Z*_*i*_ and *L*_*i*_, the latent variable and the local ancestry at locus *i*, respectively. This includes the number of causal SNPs for *Z*_*i*_, and the distribution of *β*_*i*_, the effects of the causal SNPs of *Z*_*i*_. Assuming the causal SNPs are a random sample of the variants in the segment, power is also bounded by the average difference in minor allele frequency between the two founder populations. Figure 3 presents the empirical distribution of this correlation in a simple scenario, when *Z*_*i*_ depends on 1 to 50 SNPs. Overall, ${\rho}_{L_i{Z}_i}$, increases with the number of SNPs involved and with increased homogeneity of genetic effect. For example if the *β*_*i*_ = (*β*_*i*1_, … *β*_*iK*_) are distributed around the null, the expected value of ${\rho}_{L_i{Z}_i}$ is null and a local ancestry-based test would have no power. Conversely, if the coded alleles from the causal variants tend to increase the outcome value (while the reference allele has no contribution), ${\rho}_{L_i{Z}_i}$ can be substantial (e.g. >0.2, Fig. 3b).

**Fig. 4:**
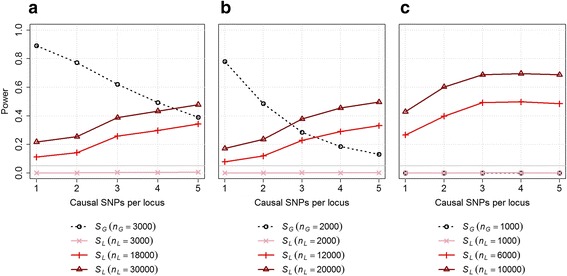
Power comparison for multiple causal SNPs per locus. Power across 25,000 replicates using a Bonferroni correction resulting in *p*-value thresholds of 1 × 10^−7^ and 1 × 10^−15^ for the local ancestry-based interaction test (*S*_*L*_) and the SNP-based interaction test (*S*_*G*_), respectively. One to five common causals SNPs were selected per interacting locus while assuming either low (**a**), moderate (**b**) or high (**c**) differentiation of those SNPs between the two admixed populations. We considered three case scenarios for the additional increase in sample size that would be achieve when using local ancestry derived from AIMs, no increase (pink), a lower bound of six fold increase (light red) and an upper bound of 10 fold increase. We varied the baseline sample size (for *S*_*G*_) across scenarios to emphasize the differences between the tests

We performed a simulation study to compare the performance of the pairwise SNP-based approach (*S*_*G*_), when using both genotyped and imputed common SNPs (MAF > 1 %), and the local ancestry approach (*S*_*L*_) while increasing *K* the number of causal SNPs per locus. For simplicity we assumed the number of causal variants was the same in the two interacting regions, and only considered common variants (minor allele frequency, MAF > 0.10). We explored scenarios where the causal SNPs were either slightly differentiated or highly differentiated between the two ancestral populations. When assuming equal sample size, *S*_*L*_ is underpowered as compared to *S*_*G*_, despite a dramatic increase in the total number of tests performed (Fig. 4). Hence when GWAS data exists, deriving local ancestry segments would be of limited interest for gene-gene interaction testing unless the number of causal variants is large (e.g. >10). However, when considering de novo genotyping with a fixed budget, an increase in sample size can be achieved when measuring local ancestry only, *S*_*L*_ can be more powerful than *S*_*G*_. In particular, assuming a 6 fold decrease in cost, *S*_*L*_ outperform *S*_*G*_ if either the differentiation is moderate or the number of causals is large (>5), or if the causal SNPs are highly differentiated (e.g. correlation between local ancestry and the causal >0.5, Fig. 4c).

We assumed in these simulations that true local ancestry is available. To evaluate the impact of additional noise introduced by ancestry inference, we analyzed the same data but using ancestry inferred using LAMP-LD. As shown in Additional file 1: Figure S1, using the inferred ancestry has only minor impact on power. This is partly expected thanks to the high accuracy of inference in African Americans [15]. The accuracy of SNP imputation could also impact the power of the SNP-based test, however the quality of imputation depends on more parameters, varying across allele frequencies and the chips used for genotyping [23]; it would therefore be more difficult to evaluate thoroughly. Instead we applied the SNP-based test using the genotyped SNPs only found on the Illumina Human1M-Duo BeadChip (Additional file 1: Figure S1). We observed a substantial decrease in power, highlighting both the importance of using imputed variants and the need for high quality imputation for the SNP-based test, which might be a concern for rare causal variants.

Finally, we evaluated how the relative power of the *S*_*L*_ and *S*_*G*_ tests is impacted when applying a two-steps procedure where SNPs and ancestry segments are first pre-selected for interaction testing based on their marginal effects [24]. For simplicity we assume that the vast majority of SNPs are not involved in interaction, so that for a *p*-value threshold *t* at step 1, the total number of interaction tests at step 2 can be approximated by $$ \left(\begin{array}{c}\hfill n\times t\hfill \\ {}\hfill 2\hfill \end{array}\right) $$, where *n* is the number of predictor (either SNP or local ancestry segment). Additional file 1: Figure S2 shows the results from this strategy when applied to a case similar to Fig. 4, but adding a main effect to each causal SNPs of the same magnitude as the interaction effect, and using *t* = 0.01 at step 1. In this specific scenario, the 2-step approach mostly benefits to the ancestry-based test, which outperform the SNP-based test in many more scenarios, including cases where sample size was the same for the 2 analyses.

## Conclusions

We explored the performance of a local ancestry-based interaction test to capture non-linear effects from two independent loci. The strategy is similar to a standard SNP-based pairwise interaction screening but uses local ancestry segments instead of SNPs. One major underlying motivation for such an approach is that the total number of tests to be conducted is dramatically lower than for a standard pairwise SNP interaction test, reducing both the computational burden and the correction for multiple testing. We demonstrate that such a test would indeed capture interaction effects between two loci as long as the individual effects of the causal variants at each locus do not cancel each other. For existing datasets that only contain local ancestry data derived from AIMs and for de novo genotyping studies looking for the optimal cost/power ratio, our approach (*S*_*L*_) can be highly relevant as it can outperform the pairwise SNP screening from standard GWAS data (*S*_*G*_). Conversely, when GWAS genotyping data does exists, in most scenarios we explored, *S*_*G*_ outperforms *S*_*L*_ when the number of causal variants at the locus was small. As the number of causals grows beyond 10, the power of *S*_*L*_ increases but does not substantially exceed *S*_*G*_ unless the differentiation of the causal SNPs between the two populations is very high. Interestingly, as the differentiation increases so does the relative power of *S*_*G*_, which explains the underperformances of *S*_*L*_. We found that, as differentiation increases, many genetic variants become good tags for local ancestry, and so *S*_*G*_ benefits from the increase in differentiation as well. Overall, the relative performances of our approach depends on the balance between the gain in power achieved thanks to the decreasing number of test and the decrease in power due to low correlation between local ancestry and the causal variant(s) at the interacting loci.

Furthermore, we used the whole genome sequence-based test (*S*_*S*_) as a reference to compare the relative performance of the two alternative approaches. While such a test might have higher power than *S*_*L*_ and *S*_*G*_ (Fig. 2), testing all possible pairs of SNPs would requires extremely intensive computational power in practice, and the implementation of such tests, which have been rarely explored to our knowledge, would require substantial software development and hardware structures (e.g. graphics processing units [25]). This confirms that, as of today, GWAS-based pairwise interaction tests remain a relevant approach for identifying interactions as compared to whole genome sequence-based approaches.

Regarding power comparison between *S*_*L*_ and *S*_*G*_, the power of the local-ancestry based interaction test was derived based on 1,000 local ancestry regions. However, the number of segments depends on the number of ancestral populations and the number of generations since admixture, and will therefore differ across admixed populations. Increase in the total number of segments can impact the correlation between local ancestry and the causal variants within these segments, as well as the total number of tests that have to be performed for an interaction screening. Using the inferred local ancestry had very limited impact on power in our simulation as the accuracy of inference is very high in African Americans. However, for other populations, the impact might be substantial. For example Brown et al. reported squared correlation between true and inferred local ancestry of 0.63, and 0.81 for Mexican and Puerto Rican population when using LANC-CSV, which had similar results to other methods [15]. While further analysis might explore such situations, we believe the results described in this study would remain valid. Finally, additional work might also include extensive explorations of scenarios where interaction factors (either single SNPs or single local ancestry segments) are selected based on their marginal association with the phenotype of interest. When applied in our simulation framework we observed a strong improvement of the ancestry-based test over the SNP-based test, however this needs to be confirmed across a broader range of scenarios.

Overall, while our approach shows some limitations when genome-wide genotypic data are available and when the number of causal variants per region is small and contains mostly undifferentiated variants, we highlight that genome-wide local-ancestry based interaction screening remains relevant. First, because some datasets only generated local ancestry data through AIMs, and do not have GWAS data. Second, considering budget constraint for de novo genotyping, and assuming a 6 fold decrease in cost for genotyping AIMs as compared to a standard GWAS, substantial additional gain in power can be achieved through local-ancestry based tests.

## Methods

### Genetic model

We considered a genetic model similar to the one described in Chatterjee et al. [26], which can be easily adapted to the local ancestry context. It consists in two independent sets of adjacent SNPs from two loci on different genomic regions, which represent in this study two local ancestry segments. Several SNPs within segment *i* have an indirect association with the outcome of interest through a latent variable *Z*_*i*_, an unmeasured quantitative biological phenotype partially governed by SNPs within the locus. Interaction effects between the genetic variants on the outcome is introduced through an interaction term between the *Z*_1_ and *Z*_2_ variables (i.e. the cumulative effect of the genetic variants within a locus depends on the cumulative effect of the variants in a distant locus). More specifically the outcome *Y* is defined as follows:

1 $$ Y={\theta}_1{Z}_1+{\theta}_2{Z}_2+{\theta}_{12}{Z}_1{Z}_2+\varepsilon $$

where *Z*_1_ and *Z*_2_ are the two latent variables that each depend on *K* SNPs and *θ*_1_, *θ*_2_, *θ*_12_ respectively represent the main effect of *Z*_1_, *Z*_2_, and the interaction of *Z*_1_ and *Z*_2_ on *Y*; *ε* is the residual noise and is normally distributed with mean 0. The latent variables *Z*_*i*_ are defined as follows:

2 $$ {Z}_i={\displaystyle \sum_{k=1}^K}{G}_{ik}\times {\beta}_{ik} $$

where *G*_*ik*_ and *β*_*ik*_ is the standardized genotype of SNP *k* in locus *i* and its main effect respectively. The SNP effects *β*_*i*_ = (*β*_*i*1_, … *β*_*iK*_) were randomly drawn from either normal or left-truncated normal depending on the scenario explored. For simplicity, as the main effect of the latent variable has no impact on the interaction test [27], we set *θ*_1_ and *θ*_2_ to 0, and set *θ*_12_ to 1. Except when specified otherwise we scaled the variance of *ε* so that the proportion of the variance of *Y* explained by the interaction equals 1 % (to reflect values observed in GWAS for common complex traits).

### A single major causal SNP per segment

We first assumed the genetic effect of a segment is driven by a single causal variant, while the effect of other potential SNPs is null or negligible. This is equivalent to assuming *K* = 1 in equation (2). The effect of the SNPs on *Y* can then be re-written:

3 $$ Y={\beta}_{G_1}{G}_1+{\beta}_{G_2}{G}_2+{\beta}_{intG}{G}_1{G}_2+\varepsilon $$

Assume *G*_1_ and *G*_2_ are tagged by *L*_1_ and *L*_2_ respectively, where *L*_*i*_ is the local ancestry measured at the segment harboring SNP *G*_i_. Note that *G*_1_ and *G*_2_ do not necessarily need to be typed to correctly identify local ancestry (local ancestry spans many MB’s in recently admixed populations and can be reliably identified using a small set of variants). For simplicity, we considered only the case of a two-way admixed population, so that local ancestry would be typically coded as an ordinal variable with value corresponding to the number of chromosomes harboring a particular ancestry. Hence, for African-American, *L*_._ equals 0, 1 or 2. When the population under study is an admixture of more than two ancestries, testing for interaction would be more complex because of additional combinations of ancestries (e.g. for 3 ancestries A, B and C, an individual can have any of the six following ancestries at a given segment: AA, AB, AC, BB, BC and CC). To our knowledge there is no established standard to handle such situations, the simplest solution consists of testing one ancestry versus the rest [17].

We compared the relative performances of the standard test of interaction of *β*_*intG*_ (equation (3)) versus the test of *β*_*intL*_ (equation (4)), the interaction effect observed between *L*_1_ and *L*_2_ on *Y*, which can be obtained from the model:

4 $$ Y={\beta}_{L_1}{L}_1+{\beta}_{L_2}{L}_2+{\beta}_{intL}{L}_1{L}_2+{\varepsilon}^{\mathit{\hbox{'}}} $$

Note that both equation (3) and (4) are standard 1 ° of freedom tests of interaction effect estimates obtained from multiple linear regression (see our R script example in Additional file 2). The only difference being that the later test uses local ancestry instead of genotyped SNPs. When the causal SNPs are available (e.g. from whole genome sequence data) and have been standardized, the Wald test of *β*_*intG*_ is defined as ${S}_S={\left({\widehat{\beta}}_{intG}/{\widehat{\sigma}}_{\beta_{intG}}\right)}^2$. Similarly the test of *β*_*intL*_ is defined as ${S}_L={\left({\widehat{\beta}}_{intL}/{\widehat{\sigma}}_{\beta_{intL}}\right)}^2$. Under the null hypothesis of no association both *S*_*S*_ and *S*_*L*_ follow a *chi-square* distribution with one degree of freedom. Let *ρ* denote the correlation between two variables. The two scores can be written as:

5 $$ {S}_S=\kern0.5em N\ast {\rho}^2\left(Y,{G}_1\times {G}_2\right) $$

6 $$ {S}_L = N\ast {\rho}^2\left(Y,{G}_1\times {G}_2\right)\ast {\rho}_{LC}^2 $$

where *ρ*_*LC*_^2^ = *ρ*^2^(*G*_1_ × *G*_2_, *L*_1_ × *L*_2_) is the squared-correlation between the true interaction *G*_1_ × *G*_2_ term and the local ancestry interaction term *L*_1_ × *L*_2_.

In another scenario, one would test for interaction effects between pairs of SNPs from a standard GWAS chip, which implies that the tested SNPs, say *G*_1_^*^ and *G*_2_^*^ are tagging the two causals, but (likely) at a higher level than the local ancestry. We denote this test *S*_G_:

7 $$ {S}_G = N\ast {\rho}^2\left(Y,{G}_1\times {G}_2\right)\ast {\rho}_{GC}^2 $$

where *ρ*_*GC*_^2^ = *ρ*^2^(*G*_1_ × *G*_2_, *G*_1_^*^ × *G*_2_^*^) is the squared-correlation between the true interaction *G*_1_ × *G*_2_ term and the GWAS interaction term *G*_1_^*^ × *G*_2_^*^. The power of the three tests can be derived as:

8 $$ {\mathrm{Power}}_{S_{.}}=1-F\left({\chi}_{1,1-\alpha, 0}^2\Big|1,{S}_{.}\right) $$

where *F*(*χ*^2^|*d*, *S*_._) is the cumulative probability function of the non-central chi-square distribution with *d* degrees of freedom and non-centrality parameter *S*_._; *χ*_*d*,*p*,0_^2^ is the inverse of *F* under the null, i.e. the quantiles of the non-central chi-square distribution, and *α* is the type I error rate. The relative performances of the three strategies can then be evaluated by comparing the sample size *N* needed to identify the interaction at the 5 % significance level (*α*) after accounting for *n*_*S*_, *n*_*L*_, and *n*_*G*_ the number of tests that has to be performed respectively in the whole genome setting, the local ancestry setting and the GWAS setting, respectively. Therefore, the alpha levels for tests *S*_*S*_*S*_*L*_ and *S*_*G*_ were set at *α*/*n*_*S*_, *α*/*n*_*L*_, and *α*/*n*_*G*_, respectively.

For the test *S*_*S*_, we assumed the true causal variants are available as part of a whole genome sequence data of *n*_*S*_ = 20M SNPs, so that the total number of pairwise test equals 2x10^−14^. We assumed a total of *n*_*L*_ = 1, 000 local ancestry segments for the test *S*_*L*_, and *n*_*G*_ = 1M SNPs for test *S*_G_. We considered values of *ρ*_*LC*_ and *ρ*_*GC*_ in the range [0.3; 1], so that the minimum squared-correlation was 0.09. A correlation of 1 corresponding to the highest potential increase in power that can be achieve, since it will be equivalent to testing the interaction with the true causals while dramatically reducing the multiple testing corrections.

### Multiple causal SNPs per segment

We used models from equation (1) and (2) and generated outcome data across 1,000 replicates while using simulated genetic and local ancestry data (*see further section on simulation*). For ease of computation, we considered the number of causal SNPs per segment to be equal between the two interacting loci. The *β*_*i*_ were randomly drawn from a left-truncated normal (cut at 0) distribution with mean 0 and variance 1. The causal alleles were chosen to be minor in one of the populations so that all effects go in the same direction in one population. We considered scenarios including 1 to 5 causal SNPs per locus, and selected the variants so that correlation between local ancestry and each SNP was either >0.1, >0.2 or >0.5, assuming low, moderate or high differentiation at the causal SNPs, respectively.

We perform both the local ancestry interaction and the pairwise SNP interaction test in a standard linear regression. However, instead of testing all combination of SNP, we first find the best combined tag in each locus (i.e. the single SNP *j* that maximizes ${\displaystyle \sum_K}\left({\beta}_{ik}{\rho}_{jk}\right)$ across the *K* causal variants at locus *i*, where *ρ*_*jk*_ is the correlation between the tag SNP *G*_j_^*^ and the causal variant *G*_*k*_) and then test only the product of that genotype with the best tag genotype at the second locus. Power was defined as the number of replicates for which the pairwise SNP interaction is significant at $$ p=0.05/\left(\begin{array}{c}\hfill {n}_G\hfill \\ {}\hfill 2\hfill \end{array}\right) $$, where *n*_*G*_ the number of SNPs tested equals 1 M when including genotyped SNPs only and equals 10 M when including common imputed SNPs from 1000 Genomes. For the local ancestry-based test, the threshold was $$ p=0.05/\left(\begin{array}{c}\hfill {n}_L\hfill \\ {}\hfill 2\hfill \end{array}\right)=1\times {10}^{-7} $$, corresponding to the test of *n*_*L*_ = 1, 000 local ancestry segments.

### Simulation of admixed populations from the 1000 genome project

Similar to previous work [15], we simulated admixed chromosomes of African-Americans as a random walk over 1000 Genomes haplotypes [22]. CEU and FIN populations were used to represent European haplotypes and the YRI population represented the African haplotypes. We assumed that between any two base pairs there was a 10^−8^ chance of recombination. At a recombination event, the next haplotype was selected with a 20 % chance of being European and an 80 % chance of being African to reflect the estimated admixture proportions in the literature [12, 28]. Haplotypes were sampled with replacement. We simulated 20,000 haplotypes each for chromosomes 19 and 20 in this manner and added them together to form 10,000 unphased genotypes and true local ancestries. Local ancestry was then inferred using LAMP-LD [13] with default settings and with the GBR and TSI populations representing Europeans and the LWK representing Africans. LAMP-LD was run only using variants found on the Human1M-Duo BeadChip.

### Availability of supporting data

All supporting data are included as additional files.

## Additional files

Additional file 1: Figures S1 and S2. Power comparison between SNP-based and local ancestry-based interaction tests when using inferred ancestry and 1 M genotyped SNPs. **Figure S2.** Power comparison between SNP-based and local ancestry-based interaction tests when using a two steps approach. (PDF 555 kb) Additional file 2:
**R script and corresponding input files to perform 1 ° of freedom interaction test between ancestry segments.** (ZIP 40 kb)

## Abbreviations

SNP: Single nucleotide polymorphisms
GWAS: Genome-wide association study
LD: Linkage disequilibrium
AIM: Ancestry informative marker
MAF: Minor allele frequency
