# Supplementary material for: Leveraging local ancestry to detect gene-gene interactions in genome-wide data
Source: BMC Genet. 2015 Oct 24;16:124. doi: 10.1186/s12863-015-0283-z (PMC4619349; doi:10.1186/s12863-015-0283-z)

Leveraging local ancestry to detect gene-gene interactions in genome-wide data

Supplemental Data

Figure S1. Power comparison when using inferred ancestry and 1M genotyped SNPs

Power of the ancestry-based interaction test (S_L) using true ancestry (plain line, red) and inferred ancestry (dashed lines, red), and for the SNP-based interaction test (S_G) using genotyped and imputed SNPs (plain line, black), or genotyped SNPs from the Illumina Human1M-Duo BeadChip, respectively. The Bonferroni correction p-value threshold was $1x10^{-7}$ for S_L and $1x10^{-15}$ and $1x10^{-13}$ for the two S_G tests, respectively. Power was derived over 25,000 replicates across three scenarios. For each replicate, one to five common causals SNPs were selected per interacting locus while assuming either low (a), moderate (b) or high (c) differentiation of those SNPs between the two admixed populations. Sample sizes was equal to 3,000, 2,000 and 1,000, for the S_G test in scenario (a), (b) and (c), respectively. Sample size was 6 times larger for the S_L tests.

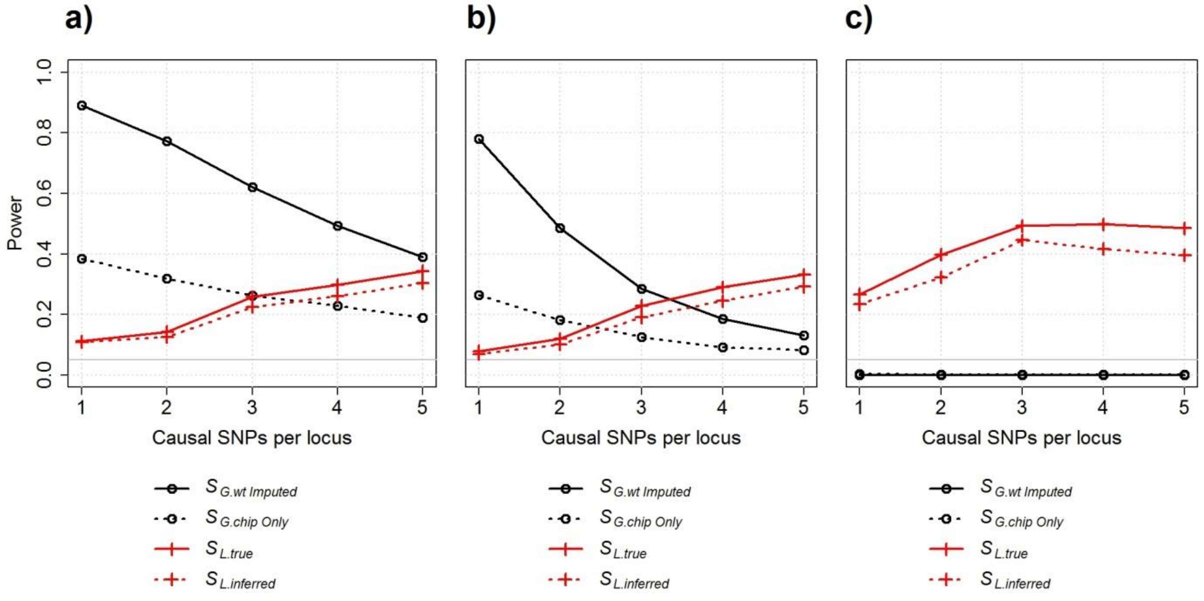

Figure S2. Power comparison for a two-step approach

Power of the local ancestry-based interaction test (S_L) and the SNP-based interaction test (S_G) when using a two-step approach where candidate ancestry segments and candidate SNPs are preselected for interaction screening if their p-values for marginal association with the outcome is below 1%. The significance threshold for the interaction effect screening at the second step was approximated assuming that most SNPs do not interact with the outcome. It equals $1x10^{-3}$ and $4x10^{-11}$ for the S_L and S_G tests respectively. Power was derived over 25,000 replicates for three underlying models. For each replicate, one to five common causals SNPs were selected per interacting locus while assuming either low (a), moderate (b) or high (c) differentiation of those SNPs between the two admixed populations. We considered three case scenarios for the additional increase in sample size that would be achieve when using local ancestry derived from AIMs, no increase (pink), a lower bound of six fold increase (light red) and an upper bound of 10 fold increase. We varied the baseline sample size (for SG) across scenarios to emphasize the differences between the tests.

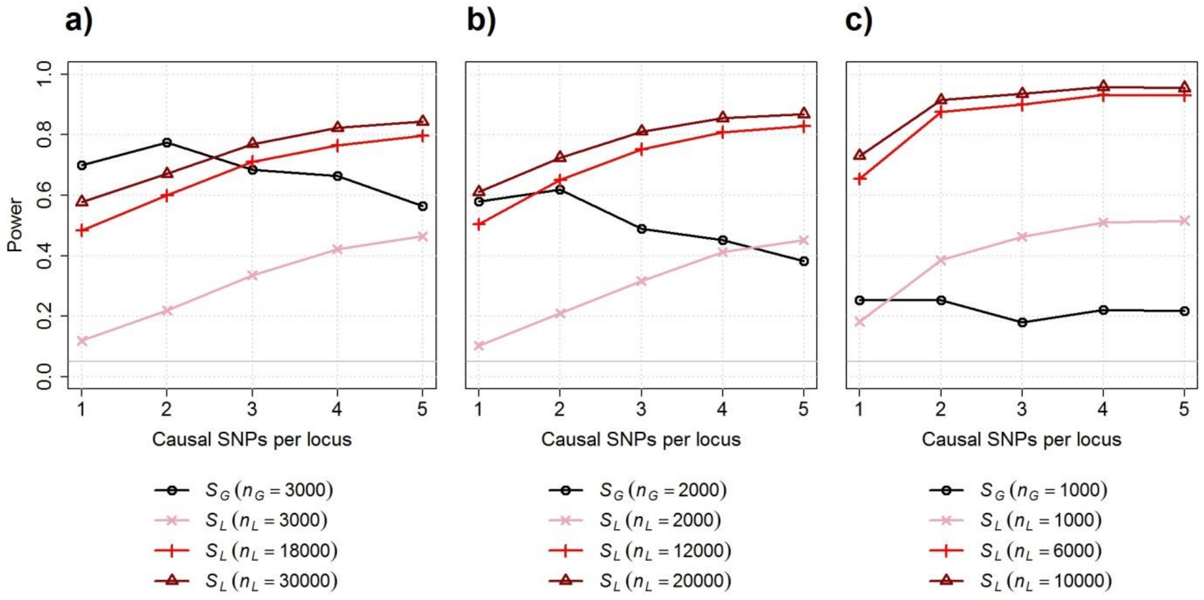

Supplement: Additional file 1: Figures S1 and S2. — Power comparison between SNP-based and local ancestry-based interaction tests when using inferred ancestry and 1 M genotyped SNPs. Figure S2. Power comparison between SNP-based and local ancestry-based interaction tests when using a two steps approach. (PDF 555 kb) [file 12863_2015_283_MOESM1_ESM.pdf]
